# Evasion of Influenza A Viruses from Innate and Adaptive Immune Responses

**DOI:** 10.3390/v4091438

**Published:** 2012-09-03

**Authors:** Carolien E. van de Sandt, Joost H. C. M. Kreijtz, Guus F. Rimmelzwaan

**Affiliations:** Department of Virology, ErasmusMC, Dr. Molewaterplein 50, 3015 GE, Rotterdam, The Netherlands; Email: c.vandesandt@erasmusmc.nl (C.E.S.); j.kreijtz@erasmusmc.nl (J.H.C.M.K.)

**Keywords:** influenza, evasion, innate immunity, adaptive immunity

## Abstract

The influenza A virus is one of the leading causes of respiratory tract infections in humans. Upon infection with an influenza A virus, both innate and adaptive immune responses are induced. Here we discuss various strategies used by influenza A viruses to evade innate immune responses and recognition by components of the humoral and cellular immune response, which consequently may result in reduced clearing of the virus and virus-infected cells. Finally, we discuss how the current knowledge about immune evasion can be used to improve influenza A vaccination strategies.

## 1. Introduction

Influenza viruses belong to the family of orthomyxoviridae and are one of the leading causes of respiratory tract infections in humans [1]. Yearly, influenza viruses cause an estimated 3–5 million severe clinical infections and 250,000–500,000 fatal cases [2,3]. The genome of influenza A viruses consists of eight gene segments of single-stranded negative-sense RNA encoding 12 proteins: surface glycoproteins, hemaglutinin (HA) and neuraminidase (NA), two matrix proteins (M1 and M2), the nucleoprotein (NP), three polymerase complex proteins PB1, PB2 and PA, and four non-structural proteins NS1, NS2, PA-X and PB1-F2 [1,4]. 

**Figure 1:**
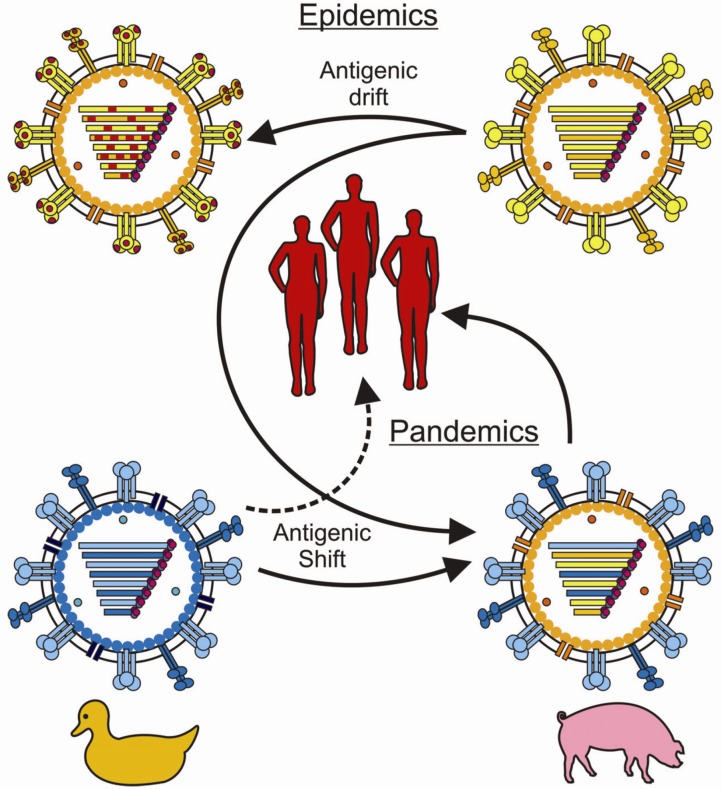
Antigenic drift and shift to escape immunity. The gradual accumulation of mutations, mainly in the highly variable globular head region of HA, causes the influenza virus to escape recognition by virus neutralizing antibodies and allows it to cause seasonal epidemic outbreaks. This phenomenon is called antigenic drift. The introduction of a novel subtype into the human population is called antigenic shift and may cause a pandemic outbreak in the naïve human population when the virus is efficiently transmitted from human to human, since antibodies directed against the novel subtype are absent. Past pandemic outbreaks were caused by exchange (re-assortment) of gene segments between two or more influenza strains, e.g., avian and human. However, recent studies in ferrets suggest that avian influenza viruses, like H5N1, could be directly transmitted from animal reservoirs into the human population, requiring only a small number of adaptive mutations [9] as indicated by the dotted line in this figure.

Influenza A viruses are subdivided, based on their surface glycoproteins; currently 17 subtypes of HA and nine subtypes of NA are known [5,6,7]. Seasonal influenza A viruses of the H1N1 and H3N2 subtype and influenza B viruses cause yearly epidemics [2]. This can be attributed to their ability to escape recognition by virus specific antibodies due to antigenic drift (Figure 1). As a result, seasonal influenza vaccines need to be updated almost annually to match the new epidemic strains and to remain efficacious [8]. 

Introduction in the human population of influenza A viruses with antigenically distinct HA molecules, including novel subtypes, is known as antigenic shift (Figure 1). When such an antigenically-distinct virus is transmitted efficiently from human to human, it may cause a pandemic influenza outbreak, since neutralizing antibodies to this virus are absent in the population at large [10]. Examples of pandemics include the Spanish flu of 1918 caused by a virus of the H1N1 subtype which killed 20–50 million people [11], the Asian flu of 1957 caused by a re-assorted H2N2 virus and the Hong Kong flu of 1968 caused by a re-assorted H3N2 virus. Each time, the pandemic virus replaced the subtype that circulated prior to the pandemic. In 1977, a virus of the H1N1 subtype was re-introduced, which did not result in a major pandemic, and this virus seasonally co-circulated with the H3N2 virus subtype until 2009. The first pandemic outbreak of the 21st century occurred in 2009 when a novel H1N1 re-assorted virus of swine origin was introduced into the human population [12]. In addition, other influenza virus subtypes are transmitted from animals (in particular swine and avian influenza viruses) to humans occasionally [13,14]. For example, in 1999, there were three isolated cases of influenza A/H9N2 virus infections in humans displaying mild symptoms only [15]. During an outbreak of highly pathogenic avian influenza A virus of the H7N7 subtype in the Netherlands in 2003, 89 human cases were reported of which one was fatal [16,17]. Larger is the impact of highly pathogenic avian influenza A/H5N1 viruses which have been transmitted on a regular basis from infected poultry to man since the first case was identified in 1997 in Hong Kong [18]. Since 2003, over 600 human cases have been reported, most of them suffering from severe pneumonia progressing to acute respiratory distress syndrome, 60% of the cases had a fatal outcome [19,20,21,22,23]. The reported case fatality rate most likely is an overestimate, since subclinical infections and mild cases are not reported [24]. So far, efficient human-to-human transmission has not been observed, although clusters of human cases have been reported [25,26,27]. Furthermore, recent studies have shown that, in principle, transmission of highly pathogenic H5N1 viruses amongst mammals is possible and that only a limited number of adaptive mutations are required for airborne transmission, emphasizing the pandemic potential of these viruses [9,28,29]. 

Without lifelong protection against seasonal influenza virus infections and the threat of possible future pandemics, it is of great importance to have insight in how immunity against influenza A infections is formed and how influenza A viruses manage to evade these immune responses. In this review, we describe the role of innate and adaptive immunity against influenza A virus infections and evasion strategies used by influenza A viruses to escape immunity. Finally, we briefly discuss the impact on influenza A vaccine development.

## 2. Innate Immunity

The primary targets for influenza viruses are the epithelial cells that line the respiratory tract and which initiate an antiviral immune response upon detection of the virus. The first line of defense is formed by the innate immune system, which is quick but lacks specificity and memory. Innate immunity is formed by physical barriers (e.g., mucus and collectins) and innate cellular immune responses [30]. Here, we outline several of the innate defense mechanisms directed against influenza A infections. 

### 2.1. Sensing Of Influenza Virus Infection by Receptors of the Innate Immune System

Influenza A virus infection results in the recognition of pathogen-associated molecular patterns (PAMPs) by pattern recognition receptors (PRRs) that initiate antiviral signaling cascades, resulting in the production of interferons (IFNs), cytokines and chemokines [31]. Three main categories of PRRs are involved in recognition of an influenza A infection and the induction of an IFN response: Toll like receptors (TLRs), retinoic acid inducible gene-I (RIG-I) receptors and nucleotide oligomerization domain(NOD)-like receptor family pyrin domain containing 3 (NLRP3) [32,33,34]. 

The TLRs are the first receptors to recognize the influenza virus infection. TLR7 is an intracellular receptor that recognizes single stranded viral RNA (ssRNA) after the ribonucleoprotein complex has been degraded inside acidified endosomes [35,36]. TLR3 is another intracellular receptor that recognizes double stranded viral RNA (dsRNA) [37]. Other TLR receptors likely to sense an influenza virus infection are TLR2 and TLR4, which are present on the cell surface and recognize viral surface glycoproteins like influenza HA and NA [34,38,39,40]. At a later stage of infection, newly produced uncapped, 5'-triphosphates bearing viral RNAs are recognized by RIG-I receptors in the cytoplasm [41,42,43,44]. 

NLRP3 is part of the NLRP3 inflammasome and is activated by dsRNA which subsequently activates caspase I, resulting in the proteolytic maturation of IL-1β and IL-18 [45]. 

The signaling cascade of all activated TLRs, except for TLR3, starts with the activation of MyD88, which subsequently can activate tumor necrosis factor (TNF) receptor associated factor 6 (TRAF6), either directly or via IL-1R-associated kinase-1 (IRAK 1), eventually leading to the activation of mitogen-activated kinases (MAPKs) and nuclear factor kappa-light-chain-enhancer of activated B cells (NF-κB). TLR3 signaling cascade starts with the activation of TRIF (TIR-domain-containing adapter‑inducing interferon-β) eventually activating NF-κB and interferon regulatory factor 3 (IRF3). TLR4 can also signal via the TRIF dependent pathway by formation of a TRAM-TRIF complex. 

**Figure 2:**
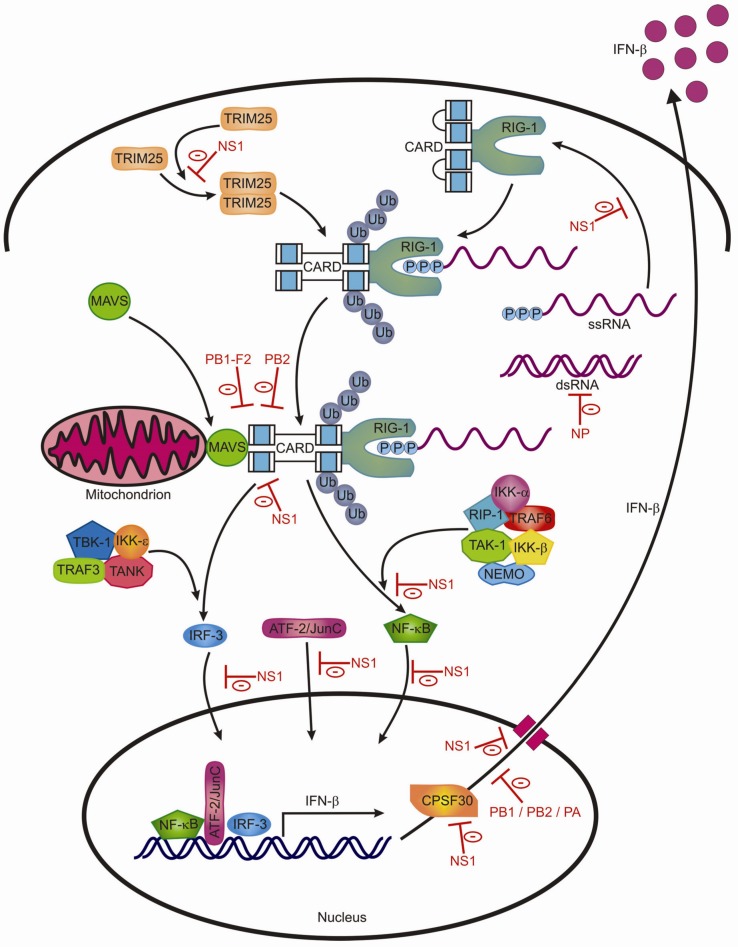
The RIG-I signaling pathway and inhibition by influenza A viruses (Figure adapted from [48]). By-products of viral replication are 5'-triphosphates ssRNA and dsRNA which can bind to the RIG-1 receptor, leading to conformational changes, causing exposure of the CARDS which are ubiqutinated by TRIM25. Subsequently, RIG-1 associates with MAVS and thereby starts a signaling cascade leading to activation of transcription factors IRF3, NF-κB and ATF-2/JunC, resulting in the transcription of IFN-β mRNA. Indicated in red are sites at which the influenza A virus interferes with this pathway, as explained in the text.

Activation of RIG-I receptor by binding of viral 5'-triphosphates RNA or viral dsRNA to the repressor domain (RD) of RIG-I results in conformational changes exposing the caspase activation and recruitment domains (CARDs). These domains are ubiquitinated by IFN-inducible E3 ubiquitin ligase, tripartite motif 25 (TRIM25) [46]. RIG-I can then associate with mitochondrial antiviral signaling adaptor (MAVS; also known as IPS-1, VISA or Cardif), starting a signaling cascade that leads to the activation of IRF3 and NF-κB. The signaling cascades via TLRs and RIG-I receptors have been extensively reviewed by others [44,47,48,49] (Figure 2). 

**Figure 3:**
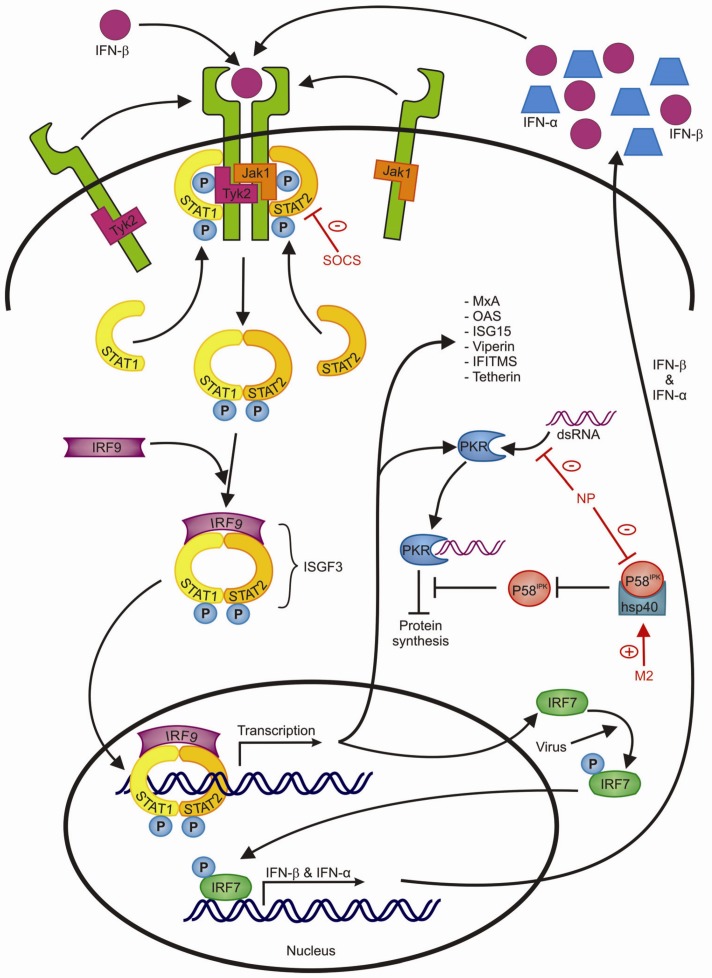
The type I IFN signaling pathway and inhibition by influenza A viruses (Figure adapted from [48]). IFN-β produced by influenza virus-infected cells binds IFN receptors causing the phosphorylation of Tyk2 and Jak1. This is followed by binding and phosphorylation of STAT1 and STAT2 which subsequently form a complex with IRF9. This ISGF-3 complex acts as a transcription factor for >300 genes, several of which display an antiviral effect (see text). The expressed protein PKR is activated upon recognition of viral dsRNA, leading to inhibition of protein synthesis, including viral proteins. PKR is inhibited by the cellular protein P58^IPK^, however P58^IPK^ activity is downregulated by binding cellular hsp40. The IRF7 protein is phosphorylated in the presence of influenza A virus, leading to activation of a positive feedback loop, causing the transcription of IFN-α and IFN-β. Indicated in red are mechanisms of the influenza A virus to interfere with this pathway, these interfering mechanisms are explained more extensively in the text.

All these pathways eventually result in the transcription of proinflammatory cytokines, chemokines and IFNs that activate the antiviral response and the recruitment of neutrophils, activation of macrophages and maturation of dendritic cells (DCs) [31]. So far, three IFN types have been identified [50]. Type I IFNs include IFN-α and IFN-β which have an important role in limiting viral replication [51,52]. Type I interferons secreted by infected cells act on IFN-α/β receptors (IFN-α/βR) of the same cell or neighboring cells, activating an antiviral signaling cascade that involves phosphorylation of tyrosine kinase 2 (Tyk2) and Janus kinase 1 (Jak1), also called, “just another kinase 1”, which then phosphorylate signal transducer and activators of transcription (STAT) 1 and STAT2. Phosphorylated STAT1 and 2 combine with IRF9 to form ISGF3 (IFN-stimulated gene factor-3 transcription factor complex) which is responsible for the transcription of >300 genes that encode for e.g., antiviral proteins (Table 1) that establish an antiviral state in the cell that limits viral replication [49] (Figure 3). IFN-β acts through a positive feedback loop on the IFN-β receptor which activates IFN stimulated gene factor 3 (ISGF3), resulting in the expression of IRF-7. IRF-7 is phosphorylated in the presence of a viral infection and induces the expression of both IFN-α and IFN-β [53]. 

**Table 1:** IFN-induced antiviral proteins and their function.

Protein	Function	Reference
MxA (Myxovirus resistance gene A)	Inhibits viral replication by interfering with the viral ribonucleoprotein structure	[54,55,56]
PKR (Protein kinase R)	Limits viral replication by blocking general translation	[57,58]
OAS (2'–5'oligoadenylate synthetase)	Stops viral replication by means of activating RNAseL which results in degradation of viral and cellular RNA and eventually apoptosis of the virus infected cell	[59,60]
ISG15 (IFN-stimulated gene 15)	Regulates a number of IFN-stimulated proteins	[61]
Viperin	Inhibits viral release by interfering with viral budding	[62]
Tetherin	Inhibits formation of influenza virus particles	[63,64]
IFITMs	Restrict viral entry	[65]

IFN-γ is the main type II IFN and contributes to the establishment of an effective adaptive cytotoxic T cell (CTL) response against the influenza virus infection. It regulates virus-specific CTL homeostasis in secondary lymph nodes and subsequent trafficking of CTLs to the site of infection [66]. Furthermore, IFN-γ plays an important role in memory CTL responses [67]. Type III IFNs, like IFN-λ, also control influenza A infections in the lung [68].

### 2.2. Macrophages

During homeostasis, alveolar macrophages exhibit a relatively quiescent state, producing only low levels of cytokines, and suppress the induction of innate and adaptive immunity [69,70]. CCL2, produced by infected epithelial cells during the initial phase of the influenza virus infection, attracts alveolar macrophages and monocytes via their CCR2 receptor [71,72,73]. Activated macrophages enhance their pro-inflammatory cytokine response, including IL-6 and TNF-α [74,75]. Alveolar macrophages have a direct role in limiting viral spread by phagocytosis of apoptotic infected cells [40,76,77] and by phagocyte-mediated opsonophagocytosis of influenza virus particles [78]. They are also involved in regulating the adaptive immune response. Depletion of alveolar macrophages prior to influenza virus infection led to a reduction of antibody titers and reduced numbers of virus-specific CTLs post-infection [77]. In contrast to these beneficial effects, alveolar macrophages also pose a negative effect, since their activation also results in the production of nitric oxide synthase 2 (NOS2) and TNF-α which contribute to the severe pathology that can be the result of an influenza virus infection [71,79,80].

### 2.3. Natural Killer Cells

Natural killer (NK) cells are cytotoxic lymphocytes of the innate immune system. They are able to lyse infected cells in a MHC class I independent manner via a direct or indirect mechanism of recognition. The sialylated NKp44 and NKp46 receptors are bound by the HA proteins expressed on the surface of influenza virus-infected cells [81]. This results in direct lysis of the infected cell [82,83]. It was shown that mice lacking the NKp46 receptor equivalent, NCR-1, displayed increased morbidity and mortality following influenza A infection [84]. NK cells with their CD16 receptor (FcγRIII) can bind to the Fc portion of antibodies bound to influenza virus-infected cells and mediate lysis of these cells. This process is known as antibody-dependent cell cytotoxicity (ADCC) [85,86,87]. 

### 2.4. Dendritic Cells

**Figure 4:**
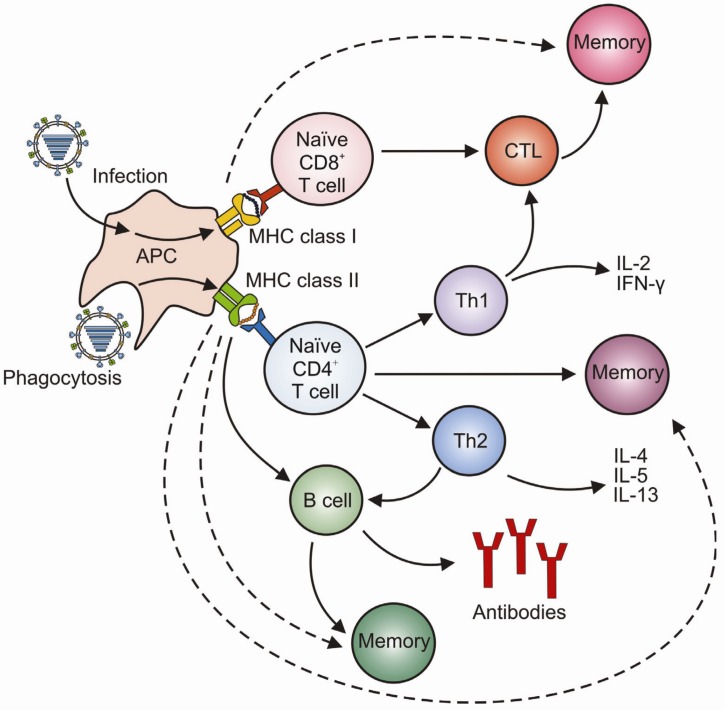
Induction of humoral and cellular immunity. Induction of immune responses after a primary influenza A virus infection is indicated by solid arrows. The more rapid activation of virus-specific memory cell populations upon secondary encounter with an influenza A virus are indicated by dotted arrows.

**Figure 5:**
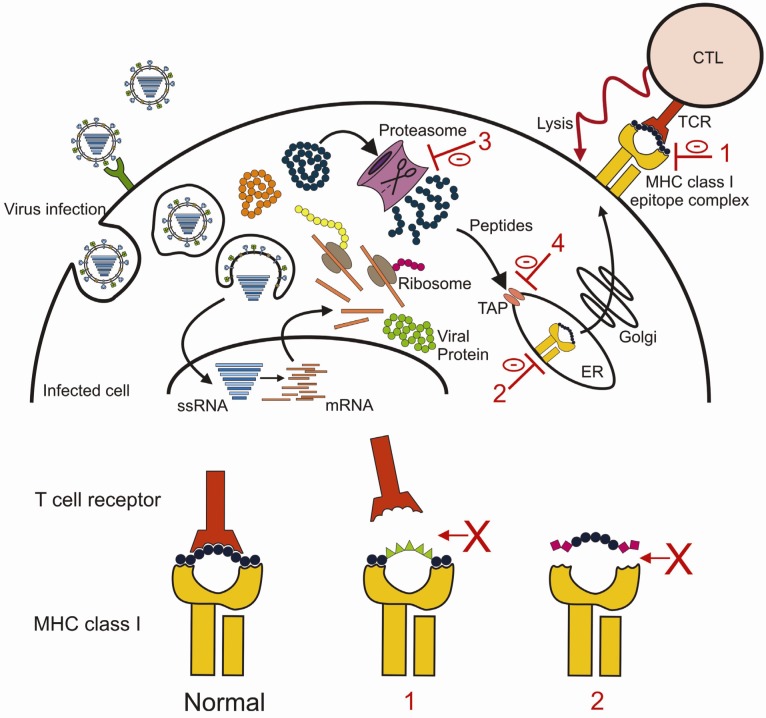
MHC class I presentation of influenza A virus epitopes and viral escape. This figure represents a virus-infected cell and the presentation of viral epitopes by MHC class I molecules. The virus can escape recognition by virus specific CTLs by: (**1**) Mutations in TCR contact residues of CTL epitopes in order to prevent recognition of the epitope MHC class I complex by specific CTLs, or (**2**) mutating the anchor residues of the CTL epitope which prevents binding of the epitope to MHC class I molecules. Furthermore, mutations outside the CTL epitope may affect antigen processing by the proteasome or transport via the TAP respectively (**3** and **4**).

Dendritic cells (DCs) are professional antigen presenting cells (APCs) which form an important bridge between the innate and the adaptive immune system. During an infection, DCs initiate adaptive immune responses by the presentation of viral antigens to naïve and memory T and B lymphocytes (Figure 4) [69,88,89]. At steady state conditions, DCs constantly survey the lungs for invading pathogens or foreign material [90]. Once the lungs are infected with influenza A virus, the DCs can acquire the antigens via two distinct mechanisms. The first route is by direct infection of DCs by influenza A virus [91,92]. Proteasomes in the cytosol degrade viral proteins into small peptides which are transported to the endoplasmic reticulum (ER) via TAP (transporter of antigen processing), where they are loaded to MHC class I molecules. These MHC class I peptide complexes are then transported via the Golgi complex onto the cell membrane where they can be recognized by virus-specific CD8^+^ cytotoxic T cells (CTLs) (Figure 5) [88,93]. The second mechanism of antigen acquisition by DCs is through phagocytosis of virus particles or apoptotic epithelial cells [88,94,95,96]. Viral proteins are then degraded into smaller peptides in endosomes/lysosomes and presented on the cell surface in MHC class II peptide complexes which can be recognized by CD4^+^ T helper cells. T helper cells assist B cells to proliferate and mature into antibody-producing plasma cells. Via this route of antigen acquisition, DCs can also present epitopes to CD8^+^ T cells. This is also known as cross-presentation. For presentation of viral antigens to virus-specific T cells, activated DCs migrate to the draining lymph nodes [90,97,98,99]. 

## 3. Adaptive Immunity

The second line of defense against influenza A virus infection is the adaptive immune response. Overall, this highly specific response is relatively slow upon first encounter with a pathogen. However, as a result of the formation of immunological memory, the response is faster and stronger after a second encounter with the same pathogen. The adaptive immune response consists of humoral (virus‑specific antibodies) and cellular (virus-specific CD4^+^ and CD8^+^ T cells) immunity. Here we summarize virus-specific recognition by components of the adaptive immune system and their contribution to clearance of influenza A virus infections.

### 3.1. Humoral Immunity

Influenza virus infection induces the production of influenza virus-specific antibodies by B cells [100,101,102,103]. In particular, antibodies directed to the viral HA and NA correlate with protective immunity. 

Antibodies directed to the trimeric globular head of HA can afford sterilizing immunity to influenza virus infection. By binding to the HA receptor binding site located in this region they can block virus attachment to host cells and/or block receptor-mediated endocytosis [104,105,106]. However, most antibodies directed against HA are influenza virus strain-specific and fail to neutralize intrasubtypic drift variants and viruses of other subtypes [6,10,107,108,109]. This is mainly due to the high variability in the HA globular head (see below). Of interest, humoral immunity elicited after an influenza virus infection does provide long-lasting antibody mediated protection against the strains that resemble the infected strain. This was exemplified during the pandemic of 2009 caused by an influenza virus of the H1N1 subtype. Elderly people that were exposed to influenza A/H1N1 virus in the 1950s had antibodies which cross-reacted with the pandemic strain and were relatively spared from contracting infections and developing disease [109,110,111,112]. Recently, broadly reacting antibodies directed against the conserved stem region of HA have been identified [113,114,115,116,117,118,119,120]. 

Antibodies to the other major glycoprotein, the viral NA, interfere with the last phase of the viral replication cycle and also exert protective immunity. NA is a sialydase and removes sialic acids from infected cells and budded virions, thereby facilitating efficient release and spread of newly formed viral particles [1]. Unlike HA-specific antibodies, NA-specific antibodies do not neutralize the virus. However, by inhibiting the enzymatic activity of NA, these antibodies limit the viral spread and thus shorten severity and duration of illness [121,122,123,124,125]. Furthermore, NA-specific antibodies may also contribute to clearance of virus-infected cells by facilitating ADCC [85].

In addition to HA and NA, influenza virus particles contain the minor glycoprotein M2. This tetrameric transmembrane protein has ion channel activity and plays an important role in unpacking the virus in the endosome [1]. A protective effect of M2-specific antibodies was first demonstrated in mice after passive transfer of M2-specific monoclonal antibodies [126,127]. M2-specific antibodies facilitate ADCC [87,128,129]. Since the M2 protein is highly conserved between various influenza A virus subtypes, M2-specific antibodies are likely to afford heterosubtypic immunity [130,131,132,133,134,135,136,137]. 

After infection, antibodies are also induced against other viral proteins, including NP [138]. Since NP is highly conserved between influenza A viruses, these antibodies could potentially contribute to heterosubtypic immunity. Although NP-specific antibodies are non-neutralizing, it was shown in mice that they contribute to protective immunity [139,140]. However, their mode of action is poorly understood, but may include ADCC of infected cells and opsonisation of NP, resulting in improved T cell responses [141,142].

After primary infection with influenza virus, serum antibodies of the IgM, IgA and IgG isotypes are induced, whereas after secondary responses, IgM responses are not observed [143]. IgM antibodies can neutralize the virus, but also activate the complement system [144,145]. In humans, virus-specific serum IgA responses seem indicative for recent infection with influenza virus [146,147]. Virus‑specific IgG antibodies correlate with long-lived protection, provided that the antibodies match the strains causing the infection [148,149,150]. In addition to serum antibodies, influenza virus infection also induces local mucosal sIgA antibody responses that protect airway epithelial cells from infection [149,151,152].

Young infants may be protected from influenza virus infection by maternal antibodies, when they match the incoming virus [153,154,155,156,157].

### 3.2. Cellular Immunity

Influenza A virus infection induces a cellular immune response, including virus-specific CD4^+^ T cells and CD8^+^ T cells. These cells play an important role in regulation of the immune response and viral clearance respectively.

#### 3.2.1. CD4^+^ T Cells

CD4^+^ T cells are activated after recognition of viral epitopes associated with MHC class II molecules and interaction with co-stimulatory molecules on APCs. Depending on the cytokine milieu, activation of naïve CD4^+^ T cells can result in the differentiation into CD4^+^ T helper 1 cells (Th1) or Th2, which can be distinguished based on their cytokine expression profiles [158]. Th2 cells produce IL-4, IL-5 and IL-13 and promote the activation and differentiation of B cells, resulting in antibody production [159,160,161]. The antibody response is strengthened by the induction of antibody class switching and somatic hypermutations affecting the variable region of the antibody, resulting in affinity maturation of the influenza-specific antibodies [162,163,164,165]. Th1 cells produce IFN-γ and IL-2 and are mainly involved in promoting CTL responses [166,167], and are essential for the induction of memory CD8^+^ T cells [168,169,170]. Memory CD4^+^ T cells, induced after a primary influenza A virus infection, contribute to faster control of subsequent influenza A virus infections [171]. Lung-resident memory CD4^+^ T cells in particular have an important role in protection against secondary influenza A virus infections [172]. In addition to helper function, CD4^+^ T cells also display cytolytic activity [173,174]. It was shown that these cells play a role in protective immunity against influenza A virus infections in humans [175]. A more extensive review on the role of CD4^+^ T cells in heterosubtypic immunity can be found elsewhere [176]. 

#### 3.2.2. CD8^+^ T Cells

Naïve CD8^+^ T cells are activated after recognition of viral epitopes associated with MHC class I molecules on APCs in the draining lymph nodes, and subsequently differentiate into CTLs. These CTLs migrate to the site of infection where they recognize and eliminate influenza virus infected cells and thereby prevent the production and spread of progeny virus [177]. Human influenza virus-specific CTLs are mainly directed against epitopes of the highly conserved internal viral proteins, like M1, NP, PA and PB2. Therefore, CTLs display a high degree of cross-reactivity with influenza A viruses of various subtypes [178,179,180,181,182,183,184]. T cell receptor (TCR) activation by a specific epitope-MHC class I complex results in a lytic response, mediated by the release of perforin and granzymes causing apoptosis of the infected cell [185,186,187]. Furthermore, proinflammatory cytokines are produced, like TNF-α, which also inhibit virus replication and enhance lytic activity [185,188,189,190,191]. Also, FasL expression is upregulated which promotes apoptosis of infected cells [187]. After infection, a pool of long-lived antigen-specific central memory and effector memory CD8^+^ T cells is formed, which form the basis for more rapid and stronger recall responses upon secondary infections [192,193,194,195,196,197,198,199,200,201]. 

Much of the current knowledge about the protective role of CD8^+^ T cells in influenza A virus infections has been obtained from mouse studies which showed that CD8^+^ T cells contribute to homo- and heterosubtypic immunity [202,203,204,205,206,207,208,209]. Evidence that CTLs protect against influenza in humans is sparse. A recent study indicated the presence of heterosubtypic memory CD4^+^ and CD8^+^ T cells against the 2009 pandemic H1N1 virus in naïve individuals [210]. It was shown that the extent of lytic activity of PBMC inversely correlated with the extent of virus shedding after experimental infection of subjects that lacked antibodies to the life-attenuated strain used for infection [211]. More circumstantial evidence for a protective role of CD8^+^ T cells in heterosubtypic influenza infections in humans stems from epidemiological studies. People who had a symptomatic influenza A infection with the H1N1 strain prior to the 1957 pandemic were partially protected from infection with the pandemic H2N2 strain [212,213]. A similar trend was found in isolated infections with the H5N1 [214]. 

#### 3.2.3. Regulatory T Cells and Th17 Cells

In addition to the activation of virus-specific CD4^+^ T cells and CD8^+^ T cells, two other cell types are activated, namely forkhead box P3 (FOXP3)^+^ regulatory T cells (Tregs) and T helper 17 cells (Th17). Tregs play an important role in balancing the immune response during infection. They control CD4^+^ T helper cell and CTL responses in order to prevent immunopathology of infected tissues [191,215,216]. Th17 cells produce IL-6 which inhibits the effects of Tregs and therefore promote T helper responses [215]. Furthermore, Th17 have a role during influenza infections in counteracting secondary bacterial infections, e.g., *S. aureus* pneumonia. Influenza A virus infections may promote secondary bacterial pneumonia by suppressing Th17 cells in an type I IFN-dependent manner [217].

## 4. Evasion of the Antiviral Immune Response by Influenza Viruses

Immune pressure on influenza A viruses forces them to adopt strategies to evade immunity. Binding of influenza viral proteins to various components of the innate immune system leads to their inhibition (Figure 2 and Figure 3) [48], whereas a combination of immune pressure in the human population and the high mutation rate of the influenza A virus leads to the generation of new virus strains that escape the existing adaptive humoral and cellular immune responses (Figure 1 and Figure 5). 

### 4.1. Escape from Innate Immunity

Influenza A viruses have adopted various strategies to evade the antiviral nature of the innate immune system.

In particular, the NS1 protein contributes to antagonizing the antiviral innate immune response. Cells infected with genetically modified influenza viruses with a non-functional NS1 gene displayed stronger IFN responses than cells infected with wild type virus. Viruses with a NS1 defect also display reduced virulence after infection of mice and pigs [218,219,220,221,222,223,224]. NS1 inhibits RIG-I receptor signaling by various means. The NS1 protein blocks the recognition of 5'-phosphorylated viral ssRNA by the RIG-I receptor [42]. More downstream of the RIG-I signaling pathway, NS1 prevents oligomerization of TRIM25 by interacting with the coiled coil domain, and so inhibits TRIM25-mediated RIG-I CARD ubiquitination which is essential for downstream signaling [225]. Finally, activation and nuclear translocation of IRF-3, NF-κB and ATF-2/c-Jun is also prevented by NS1 [226,227,228,229]. Hereby NS1 limits RIG-I mediated transcriptional activation of the IFN-β promoter [230,231]. NS1 also alters host cell gene expression by binding to CPSF30 (cleavage and polyadenylation specificity factor); it prevents polyadenylation of the 3' end of host pre-mRNA [232,233,234]. Furthermore, NS1 limits gene expression in general, interfering with the mRNA export machinery [235,236].

NS1 is not the only viral protein that restrains the innate immune system. Both influenza PB2 (especially variants containing an aspartic acid at position 9) and PB1-F2 (only variants containing a serine at position 66) limit the production of IFN-β through association with MAVS [237,238,239,240,241]. 

Viral proteins PB2, PB1 and PA form the influenza polymerase complex, the main function of which is viral RNA and mRNA synthesis. In addition, it is also involved in cap-snatching of host mRNAs and thereby reduces host cell gene expression including that of IFN-β [242,243,244,245,246]. 

The recently discovered PA-X viral protein is able to repress cellular gene expression, especially those genes involved in regulating the initiation of the cellular immune response [4].

As described above, influenza A virus infection leads to the production of antiviral PKR (Table 1). In order for PKR to limit viral replication, it first needs to be activated by viral dsRNA. PKR activation is under tight regulation of the cellular p58^IPK^ protein which inhibits PKR activity, but is inactive when it forms a complex with heatshock protein 40 (hsp40) [247,248]. Binding of NP to the p58^IPK^-hsp40 complex releases p58^IPK^, and thereby NP inhibits the effects of PKR [249]. In contrast, the influenza M2 protein, which also binds the p58^IPK^-hsp40 complex, inhibits p58^IPK^ release and thereby limits protein synthesis which eventually leads to host cell apoptosis, possibly enhancing viral particle release [250]. 

By encapsidating influenza A viral RNA, the NP protein is likely to reduce the formation of dsRNA, which could otherwise lead to activation of RIG-I signaling. Since most PRRs are located inside the cytoplasm, the nuclear replication strategy of the influenza A virus also prevents the recognition of viral RNA by cytosolic PRR. 

In addition to limiting the production of type I IFNs, influenza A virus also disturbs type I IFN receptor signaling. Influenza A virus infection induces the expression of SOCS (suppressor of cytokine signaling) proteins which inhibit IFN α/β receptor signaling on the level of JAK/STAT activation [251,252]. 

Besides interfering with innate signaling, influenza A viruses are also able to counteract cells of the innate immune system. For example, influenza virus infection of monocytes impairs their ability to differentiate into mature DCs [253]. Furthermore, it was shown that NS1 can inhibit DC maturation, and so indirectly limit the induction of virus-specific CD8^+^ T cell responses [254]. The NK response elicited during an infection is also evaded by the influenza A virus [255]. The gradual mutation of glycosylation sites of influenza virus HA proteins leads to reduced NK recognition of the HA on virus-infected cells [256]. Downregulation of the ζ chain of NKp46 receptors by free HA proteins results in impaired signaling and thereby decreased cytotoxicity of NK cells [257]. Furthermore, influenza A virus can directly infect and kill NK cells [258]. 

### 4.2. Escaping the Humoral Immune Response

Various mechanisms contribute to immune evasion of influenza A viruses from the humoral immune response. Due to the lack of proofreading activity, the transcription of viral RNA by the viral RNA polymerase is error prone and results in mis-incorporation of nucleotides. As a result, quasi species of viruses are formed with random mutations in the genome. Under the selective pressure of antibodies that are present in the human population, induced after influenza virus infections and/or vaccination, variants are positively selected from the quasi species that have accumulated amino acid substitutions in the antigenic sites of HA that are recognized by virus-neutralizing antibodies. This phenomenon is known as antigenic drift and allows the virus to evade recognition by antibodies and to cause recurrent influenza epidemics yearly (Figure 1) [8,10,259]. 

Introduction of influenza A viruses of a novel antigenically distinct subtype into the human population is known as antigenic shift and may cause a pandemic outbreak, since neutralizing antibodies against the new virus strain are absent in the population at large (Figure 1) [10]. Introduction of antigenically distinct viruses can occur after zoonotic transmission. However, in most cases, pandemics were caused by viruses that had exchanged gene segments between human and avian or swine influenza A viruses [260,261]. For re-assortment to take place, cells need to be infected with two influenza A viruses simultaneously [262]. Since epithelial cells of the swine respiratory tract have receptors for both avian and human influenza A viruses, this species can serve as a mixing vessel for the emergence of re-assorted influenza A viruses [263,264,265,266,267]. The 1957 A/H2N2 pandemic was caused after re-assortment of human and avian influenza viruses, as was the 1968 A/H3N2 pandemic virus. The virus that caused the 2009 A/H1N1 pandemic emerged after multiple re-assortment events between avian, swine and human influenza A viruses [12,261,268,269,270,271]. The emergence of the 2009 pandemic strain highlights the importance of pigs as mixing vessels for influenza A viruses. A recent study suggests that besides the pig, the quail could also serve as a mixing vessel for emerging re-assorted influenza A viruses [272].

Of interest, functionally important and conserved sequences in the surface proteins, like the fusion peptide, are inaccessible for antibody recognition, since they are buried inside the protein [273]. Similar strategies to evade antibody recognition are shared by other viruses, like human immunodeficiency virus (HIV) [274,275].

### 4.3. Escaping the Cellular Immune Response

Viruses have adopted various strategies to evade recognition by virus-specific T cells. For example, viruses with a large DNA genome (e.g., herpes viruses) can encode proteins that interfere with various steps in the antigen processing and presentation pathways [276]. Most RNA viruses, including influenza viruses, have relatively small genomes and limited coding capacity. However, they can evade recognition by T cells through their high mutation rates and the selective pressure exerted by virus‑specific T cells. 

Relatively more non-synonymous mutations are observed in the CTL epitope regions of influenza virus NP than in the rest of the protein, indicating that CTL epitopes are under selective pressure [277]. However, mutations flanking CTL epitopes may also affect the liberation of antigenic peptides from the protein by the proteasome or transport by TAP into the ER (Figure 5) [278,279,280], as was demonstrated for HIV [281].

Amino acid substitutions inside CTL epitopes may affect presentation of the epitope in different ways. Amino acid substitutions at an anchor residue may result in complete loss of the epitope, since it may no longer bind to its corresponding MHC class I molecule (Figure 5) [282,283,284,285,286]. Mutations at TCR contact residues can affect recognition by specific T cells, since the epitope no longer matches the specificity of the TCR (Figure 5) [283,284,287,288]. These types of mutations have been observed in escape mutants of viruses that chronically infect their host, like HIV-1 [289,290]. Both types of amino acid substitutions also have been observed during the evolution of seasonal A/H3N2 influenza viruses [283,285,286]. An example of a mutation at an anchor residue is the R384G substitution in the HLA‑B*2705 restricted NP_383-391_ epitope [285]. This substitution considerably affected the human virus-specific CTL response *in vitro* [282]. It is remarkable that this mutation reached fixation rapidly, despite the fact that it is recognized by a minority of human subjects only. This could be explained by strong intra-host advantages and founder effects in a theoretical model [291].

An example of amino acid variation at TCR residues includes that observed in the HLA-B*3501 restricted NP_418-426_ epitope [287,288]. Variation in this epitope displays signs of antigenic drift [292], and dictates the specificity of the CTL response to this epitope and also forms an explanation for cross‑reactivity of CTL against contemporary viruses with historic strains [293]. Of interest, functional constraints may limit variation in CTL. For example, the M1_58-66_ epitope from the matrix protein is highly conserved, despite its immunodominant nature and its restriction by HLA-A*0201, which has a high prevalence in the human population. Influenza viruses do not tolerate mutations in this epitope without loss of viral fitness [277,294].

The R384G mutation in the NP_383-391_ epitope was also detrimental to viral fitness and was only tolerated in the presence of functionally compensatory co-mutations [295,296]. At present, it is unknown if influenza viruses can also accumulate mutations flanking CTL epitopes in order to prevent efficient processing and presentation of these epitopes. Of interest, amino acid variation in the HA of influenza A/H3N2 viruses was also associated with escape from recognition by CD4^+^ T cells, but not with escape from recognition by antibodies [297]. 

## 5. Implications for Vaccine Development

### 5.1. Current Influenza Vaccines

Currently used seasonal influenza vaccines are predominantly inactivated vaccine preparations. Their use aims at the induction of strain-specific antibodies that match the epidemic strains [298,299]. Although these vaccines are considered safe and efficacious, they also have some drawbacks that are addressed by new vaccine technologies [300]. As described above, antigenic drift of influenza viruses allows the seasonal viruses to escape the neutralizing activity of antibodies induced by previous infections or vaccination. Therefore, the vaccine fails to afford life-long protection and needs to be updated almost annually [301]. Furthermore, the production of the vaccine takes several months, so the recommendation for the vaccine strains of the upcoming influenza season is made months in advance [301]. In most influenza seasons, the predicted vaccine strains match the epidemic strains. Occasionally however, a predicted influenza vaccine strain does not match the circulating strain, resulting in suboptimal protection afforded by the vaccine [302,303,304]. In the event of an emerging pandemic outbreak, the time needed to produce and distribute a pandemic influenza vaccine is also a major drawback [305,306,307]. Seasonal influenza vaccines do not afford protection against pandemic strains of novel subtypes, since the vaccine-induced antibodies do not cross-react and cross-reactive CD8^+^ T cell responses are induced inefficiently. 

Alternatively, cold-adapted live-attenuated seasonal influenza vaccines are used [308,309,310,311]. The advantage of live-attenuated vaccines is that they also elicit cellular immune responses [312,313] and mucosal immunity [310]. More recently, live-attenuated influenza vaccines have been developed by disrupting NS1 activity through deletion or truncation of the NS1 gene [221,223,224,228,314]. Hence, the virus is unable to interfere with the innate immune response. 

### 5.2. Novel Vaccines

Ideally, seasonal vaccines are used that induce broad-protective immunity against drift variants and potentially pandemic viruses of novel subtypes. Currently-used inactivated vaccines may even interfere with the induction of cell-mediated immunity otherwise induced by natural infections, especially in young children that are still immunologically naïve to influenza viruses. In this way, inactivated vaccines can hamper the development of heterosubtypic immunity [315,316,317,318,319]. Thus, the development of vaccines that induce broadly neutralizing antibodies and preferably long-lasting heterosubtypic CTL responses is desirable. 

Since viral proteins like NP and M1 are highly conserved, they are likely targets for the induction of cross-reactive T cell responses [320]. Induction of efficient CTL responses depends on effective endogenous antigen processing and presentation by MHC class I. This requires effective delivery of viral proteins into the cytosol. Several cytosolic delivery vaccine candidates are now under investigation, including DNA vaccines, recombinant viral vectors, ISCOMS and virosomes, some of which already have made it into clinical trials [321,322,323,324,325,326,327]. Also, the induction of cross-reactive antibodies has attracted attention in recent years; antibodies directed against the more conserved stem region of the HA molecule are of especial interest. In contrast to the subtype-specific antibodies induced against the globular head of HA, these HA stem-specific antibodies display broad-neutralizing activity against multiple influenza virus subtypes [113,114]. Using this stem region as an immunogen, broadly protective antibody responses could be induced [117,328]. 

Also, the ectodomain of the M2 protein is highly conserved and antibodies induced against this region afford protection against challenge infection [133,136,137,329,330]. The mode of action is not neutralization *per se*, since M2 is a minor antigen on virus particles. However, since it is also expressed on the surface of infected cells, ADCC is probably responsible for the protective effect of these antibodies [135]. 

Thus, vaccines that induce both humoral and cell-mediated immune responses directed to conserved regions of influenza A virus, in addition to strain-specific antibodies, are likely to afford protective immunity to a large variety of influenza A viruses, including drift variants and viruses of novel subtypes.

## 6. Concluding Remarks

Our knowledge of the complexity of interaction between host immune responses and variable pathogens, like influenza viruses, has increased tremendously. Insight has been obtained on how influenza A viruses evade recognition by components of the immune system. Although these insights have helped us to decrease both morbidity and mortality, mainly by developing effective seasonal vaccines, there are still gaps in our understanding that provide room for improvement for the development of more broadly protective vaccines. The induction of CTL responses to conserved epitopes, preferably those that are under functional constraints, may be a venue to develop broadly protective vaccines. Current research also focuses on cross-neutralizing antibody responses directed to more conserved regions of the surface proteins. If these regions are also under functional constraints, vaccines aimed at the induction of antibodies against these regions would suffer to a lesser extent in terms of variability in the target region.

Collectively, the new developments may counteract the variable nature of influenza viruses and yield vaccines that afford protection against both seasonal influenza viruses and future pandemic strains. 
